# Bibliometric and thematic analysis of articles in the field of infertility (2011-2015)

**Published:** 2017-11

**Authors:** Fatemeh Makkizadeh, Farzaneh Sa'adat

**Affiliations:** * School of Social Sciences, Yazd University, Yazd, Iran.*

**Keywords:** Bibliometric, Thematic structure, Infertility, PubMed database

## Abstract

**Background:**

Infertility is a disease that results in the abnormal functioning of the male or female reproductive system. Systematic research planning on any subject, including infertility is in need of solid data regarding previous efforts in this field and to identify the gaps in the research.

**Objective:**

The aim of this research is to study the thematic structure of articles related to infertility.

**Materials and Methods:**

In this descriptive-analytical study with a scientometric approach, the PubMed database was searched for research publications indexed under "Infertility" over the period 2011-2015. Specific parameters were retrieved from the PubMed. Articles about infertility were analyzed regarding the journal of publication, topics, and countries using Net draw, Ucinet and RavarPreMap software. Also, the most influential topics were analyzed by indicators in the analysis of the network: closeness centrality, and between centrality

**Results:**

The growth in scientific productions the area of infertility over the mentioned period shows an upward trend with the highest growths seen in countries like the United States, the UK, Netherlands, China, and Germany. Moreover, the contents such as fertilization in vitro, adverse effects, spermatozoa, pregnancy rate, and treatment outcome were among the most frequently-used topics in the sphere.

**Conclusion:**

Thematic analysis can provide us the research topics, important expressions, and the relationships between them. Users and policymakers can also have a better understanding of the research status in the sphere and consequently, they can plan to increase the quantity and quality of scientific productions in a more efficient way.

## Introduction

Infertility is an inability that results in the abnormal functioning of the male or female reproductive system (1). From the medical viewpoint, infertility would affect the couple's life, work, health, personality, identity and quality of life, etc. (2). Infertility rates have hardly changed over the past 20 years, according to a new study (3, 4). 

Research on "infertility" is among the areas of expertise where researchers and scholars attempt to improve the current situation. It is critically important to access data on different aspects of the area since they are the most significant resources which will help realize scientific and cultural advancements. 

Based on different classifications, one of the topics in scientometrics is to study the structure of science and its dynamics. Scientific maps -in the format of graphic figures- can break up diverse scientific areas. They can also represent the relationships between these areas and help to understand the structures of sciences (5). A scientific map is interpreted as an analysis of publications in a scientific area through different perspectives and mapping a general approach in that area. Based on such maps and identifying developments and evolutions, areas with the most and the least closeness will be differentiated (6). 

One of the suitable tools to study the process of research in specialized areas is to focus on research and scientific articles. In the area of infertility, numerous studies have been conducted and there are many scientific productions in this sphere (7-9). There are analytical instruments in this area used to assess the studies one of which is co-occurrence word analysis. The co-words analysis, as a scientometric method for different studies, helps us to study and identify conceptual relationships between scientific texts and use such relationships to make general policies as well as to choose research topics.

Based on what was expressed, the current study tries to assess the articles published in PubMed database related to infertility using co-words analysis and also scientific mapping. As a result of this method, different objectives can be met including; topic analysis, scientific decision-making and policymaking, developing this area, implementing further research, and managing knowledge and science. 

Reviewing texts on co-word analysis and topic clustering reveals that various studies have addressed this issue from a different aspect and have examined various fields of science through this method, Including; medicine (10), integrative and complementary medicine (11), anticancer (12), treatment of depression (13), etiology of colon cancer (14) and addiction (15), social work (16), electrically conductive nanocomposites (17), complementary medicine (18), creativity (19), cancer treatment (20), and life cycle (21). 

Reviewing background shows co-word analysis is an appropriate method to map the structure of science and to create thematic maps in different areas. This research seeks to take an analytical approach and identify research areas and thematic maps of infertility on PubMed during 2011-2015 using co-word analysis and analysis of social networks. It also tries to study the growth process in topics for the conducted researches in the area. In addition, this study will introduce the countries and Journals that produced documents on PubMed database in infertility area.

## Materials and methods

The current research is of the descriptive analytical type which deals with the thematic analysis of articles with a scientometric approach. The search for articles to be included in this study was carried out on December 2016, using the PubMed database (http://www.ncbi.nlm.nih.gov/pubmed). PubMed was chosen because it is the most widely used database in medicine. The search strategy was the following: "Infertility [MH] AND journal article [PT] AND 2011:2015 [DP]" where MH stands for “Medical Subject Headings,” DP “Date of Publication”, and PT “Publication Type” “Journal Article” includes the following publication types: journal articles, introductory journal articles, and reviews. MeSH is the National Library of Medicine’s controlled vocabulary thesaurus and consists of sets of terms named “MeSH terms” arranged in a hierarchical structure (called a MeSH tree) with more specific terms arranged beneath less specific terms. 

In other words, due to the fact that required data were gained on PubMed database by searching the heading mesh; validity and reliability of the instruments used in this research are confirmed. After retrieving data, at first, bibliometric methods were applied to find the distribution of the publications within years, the name of journals, and countries. At the second stage all the articles were extracted (as many as 6,424 records), the keyword yielded from articles were introduced in PubMed were smoothed with check tags and stop word lists. For instance, adult, animal, child, etc. which were among the check tags were eliminated from keywords. The keywords which were not of contexts (for example, analysis) eliminated from descriptors. In the next stage: based on Bradford's law, as many as 76 keywords with frequencies of more than 202 were identified and selected as keywords. These keywords were taken as the main concepts based on which further analyses of the research would be implemented. Following identification of basic topics, the symmetrical co-occurrence matrix of the topics was created using RavarPreMap software. 

The symmetrical co-occurrence matrix is a square which shows a topic is common with other topics in how many articles. The number of rows and columns in this matrix are equal the number of selected concepts. Moreover, every entry in the matrix resembles the number of times when two keywords for a row and column appear in the same document. Therefore, such matrix is a symmetrical one. The entries on the main diagonal of the co-occurrence matrix equal the total number of frequencies for that keyword in the document. Table I is an example of a five-by-five matrix. The intersection of row and column is the frequency of co-occurrence for the two expressions. For instance, if we take "Spermatozoa" from the third row of the following table and take "Treatment Outcome" from the fifth column of the same table; we would witness that the resultant number would be 89 which means there are 89 documents which contain both topics. 

Analyses of data yielded from the maps were completed using formed co-occurrence matrix and also through thematic mapping in the area using Ucinet and Netdraw software. The crucial indicators in the analysis of the network are classified into three categories: closeness centrality, betweenness centrality, and degree centrality.

**Degree centrality**

It is considered as the simplest type of centrality where the value of each node will be calculated by counting the number of its neighbours. The number of neighbours is yielded based on the connectors which adjoin the node. Such measure is defined by the number of direct connections in an operator. The degree centrality of node k (pk) is calculated through the following formula:$C_{D}\left(P_{k}\right)=\sum_{i=1}^{n}a(p_{i},p_{k})$, where n is the number of nodes in the network, while $\left(p_{i},p_{k}\right)$ equals 1 if the two nodes$p_{i}$ and $p_{k}$ are connected, otherwise, it would be nil.

**Betweenness centrality**

The betweenness index of a node is the number of times the node is located on the shortest possible distance between two other nodes in the network. The nodes with high betweenness play a crucial role in connecting the networks and have a pivotal status in the network. They also play an important role in the flow of information across the network. The centrality betweenness indicator k (pk) comes from the following formula:$C_{B}\left(p_{k}\right)=\sum_{i=1}^{n}\frac{g_{\mathrm{ij}}(p_{k})}{g_{\mathrm{ij}}}:i\neq j\neq k$, where$g_{\mathrm{ij}}$ is the shortest distance between the connections of pi and pj while$g_{\mathrm{ij}}(p_{k})$ is the shortest distance between pi and pj which passes over pk.

**Closeness centrality**

Closeness centrality of a node is the average length of the shortest distances between the nodes of a network. The nodes with higher closeness index are more effective in the network and play a more pivotal role while they are more accessible to other nodes. The closeness index of the node k (pk) will be calculated by the following formula:$C_{B}\left(p_{k}\right)=\sum_{i=1}^{n}d{\left(p_{i},p_{k}\right)}^{-1}$, where (pi, pk) is the shortest distance that connects the two nodes pi and pk) 22).

**Statistical analysis**

Analysis of social networks was carried out by UciNet software 6. It contains network analytical tools, such as centrality measures and so on. Integrated with UCINET is the NetDraw program which was used to draw thematic maps. Descriptive statistical analysis of the data was done using Excel to draw charts.

**Table I:** A five-by-five sample matrix in the infertility area

	**Fertilization in vitro**	**Adverse effects**	**Spermatozoa**	**Pregnancy rate**	**Treatment outcome**
Fertilization in vitro	1956	356	114	672	438
Adverse effects	356	1624	133	214	268
Spermatozoa	114	133	1484	135	89
Pregnancy rate	672	214	135	1430	442
Treatment outcome	438	268	89	442	1314

## Results

In total, we found 6424 articles in the field of infertility indexed in PubMed through 2011-2015. Table II displays, the top 5 countries that published the articles in the field of infertility 2011-2015. The countries with the most published papers were the USA (n=2328), followed by England (n=1500), Netherlands (n=594), China (n=418), and Germany (n=389). These countries had published 33.49% of articles. Output publication of this study was published in 1011 different journals. Table III displays, the top 5 journals that published papers on the field of infertility 2011-2015. 

About 29.04% of PubMed papers were concentrated in five journals; Fertility and Sterility, Andrologia, Journal of Assisted Reproduction and Genetics, Human reproduction, and Reproductive biomedicine online.

**Bibliometric analysis**

**Figure 1:**
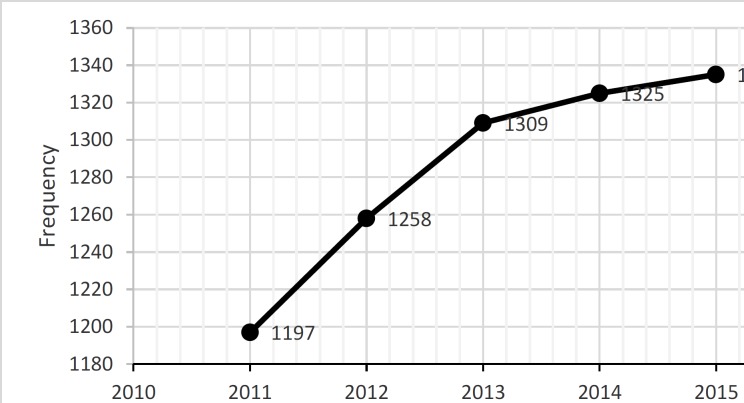
Trend of scientific productions in infertility area over the period 2011-2015.

Figure 1, presents trends in infertility studies conducted by global researchers. It shows that the number of scientific papers increased steadily from 2011 to 2015. The growth pattern of literature represents the slow development of publications from 2013 to 2015. 1197 records were published in 2011, 1258 records in 2012, 1309 records in 2013, 1325 records in 2014, 1335 records in 2015. The growth pattern of literature represents the slow development of publications from 2011 to 2015.

**Figure 2:**
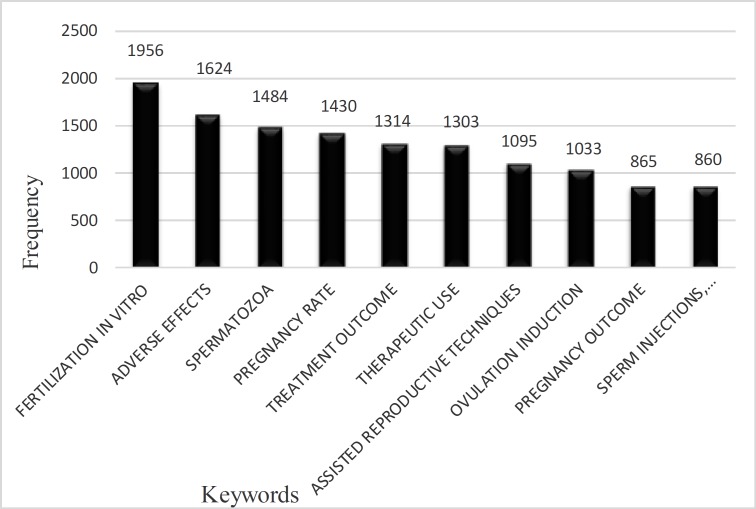
Top 10 frequently-used keywords in infertility area over the period 2011-2015.

The most frequently-used words or the most active research fields in this area over the period 2011-2015 were identified using Bradford's law. Considering the fact that keywords are considered as indicators and can highlight the topic, Bradford's law shows the thematic distribution of the articles. Based on Bradford's law, keywords are classified into three types: core, close to the core, far from the core. The first and the second types were identified as the most frequently used in this research including 239 Key words in this section, in order to refrain from making the diagram overcrowded and to help the keyword tags represent efficiently; only 10 frequently-used keywords (Figure 2) were taken. 

According to figure 2, frequently-used words include concepts such as fertilization in vitro, adverse effects, etc. A total of 66 countries were involved in creating thematic documents of the area among which the United States topped the list with 36.23%, followed by England with 23.34%, the Netherlands with 9.2%, China with 6.5%, and Germany with 6.05%. The United States alone has produced 36.23% of the global knowledge in this discipline. Meanwhile, only five countries have been mentioned to refrain overcrowding in table II, in which Iran ranks 21st with a frequency of 34 in producing infertility content. 

**Thematic analysis**

To prepare the for the analysis of social networks used UciNet software 6. It is a comprehensive package for the analysis of social networks. It contains network analytical tools, such as centrality measures and so on.Integrated with UCINET is the NetDraw program that used for drawing diagrams of social networks.

Of articles related to infertility, the entire articles extracted from PubMed were processed and then as many as 5,911 keywords were yielded. It is expected that there are averagely at least five keywords in each article. Since the keywords might repeat in multiple articles, the number of keywords would decrease but the frequency of keywords would increase. Of all the keywords processed, the ones with high frequencies were taken and then the co-occurrence map between these keywords was established. 

**Figure 3:**
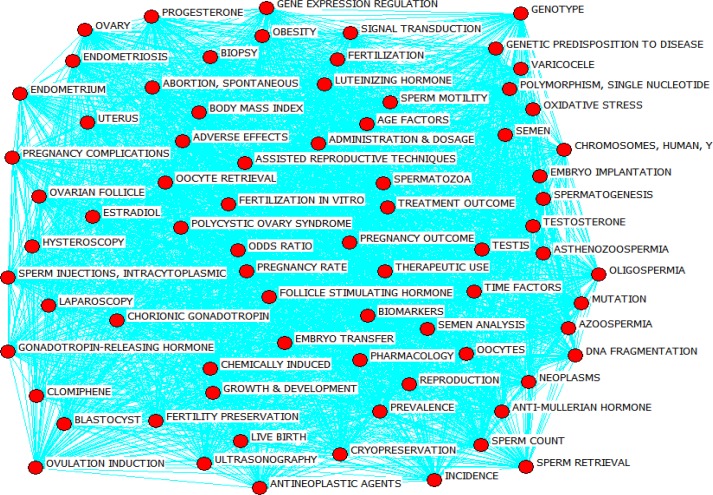
General overview of co-occurrence infertility words network over the period 2011-2015.

Figure 3 represents co-occurrence network related to infertility over the period 2011-2015. In the figure, every circle represents the keywords, while the lines show the nature of the relationship between them. A co-occurrence network is composed of nodes (keywords) and ties (the relationship between the keywords). Since the ties predominantly outweigh the nodes in number, the mapped network is of a continuous type. Since it can be seen in the figure, co-occurrence network is only composed of a big network. The ties of the network suggest that the majority of keywords link together directly or through inductors. 

**Figure 4:**
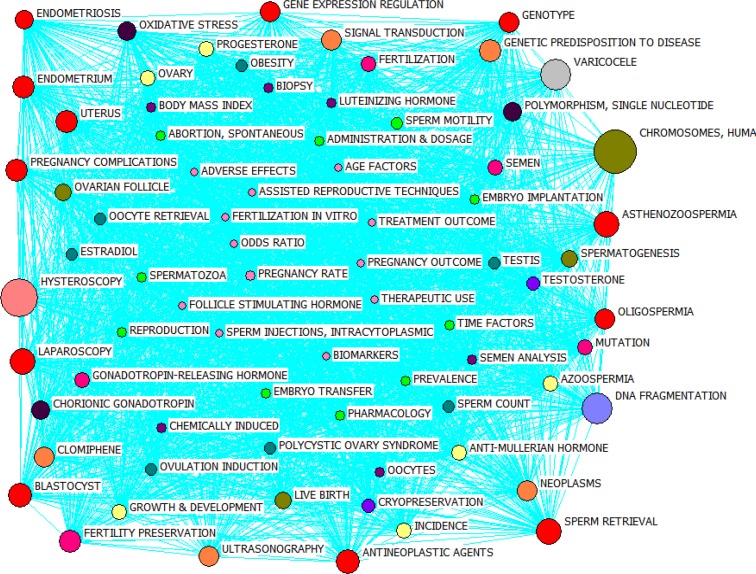
General overview of co-occurrence word network from 2011 to 2015 based on closeness centrality measure.

In order to yield a more efficient analysis of co-occurrence word network of infertility articles, the frequently-used keywords with closeness centralities other than zero were identified and co-word maps were prepared for them, which can be seen in figure 4. The diagonal of nodes represents closeness centrality. In other words, the smaller the nodes' diagonal, the closeness centrality is higher and vice versa. In the figure, circles with the same colors have equal closeness centralities and have been put together based on their closeness centralities. 

The results achieved by analyzing closeness centrality in studied articles suggest that thematic topics can be divided into three groups: first, topics such as "fertilization in vitro, adverse effects, assisted reproductive techniques, follicle stimulating hormone, etc." with closeness centrality of 74.000; sec, topics like "embryo implantation, reproduction, pregnancy rate, etc." with closeness centralities of 75.000; and third, "body mass index, oocytes, semen analysis, etc." with closeness centrality of 76.000. 

Table IV show cases keywords with the highest betweenness centralities among the entire keywords studied in the mentioned period, which fall in the first three types. 

Betweenness centrality measures the percentage of the number of shortest distances in a network that pass through the relevant node. If the value of betweenness centrality is less than 0.1, the node has no role. But if the value exceeds 0.1, then the node is the turning or central point. High values of betweenness centrality in scientific maps represent the value of the node. For instance, if a node links two irrelevant clusters, it is of great value in terms of betweenness centrality and if such a valuable node is eliminated, information flow might be interrupted in a network (23). 

**Figure 5:**
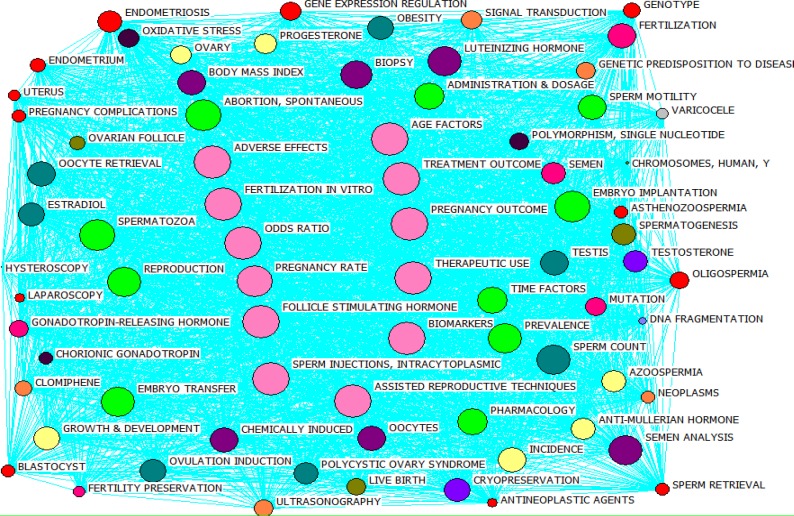
General overview of co-occurrence word network over the period 2011-2015 based on betweenness centrality measure.

The results from analysis of betweenness centrality show that thematic topics such as "fertilization in vitro, adverse effects, assisted reproductive techniques, follicle stimulating hormone, etc." (The pink circles) with betweenness centrality of 4.112 are ranked first, followed by others such as "spontaneous, abortion, and more," with betweenness centrality of 3.999, and finally spermatozoa with betweenness centrality of 3.979. In fact, the mentioned keywords have formed the shortest possible distance between the two nodes. Figure 5 represents a general overview of co-occurrence word network based on betweenness centrality measure.

**Table II:** Top 5 countries that contributed in producing scientific articles on infertility over the period 2011-2015

**Number**	**Country**	**Frequency**	**Percentage**
1	United states	2328	36.23
2	England	1500	23.34
3	Netherlands	594	9.2
4	China	418	6.5
5	Germany	389	6.05

**Table III:** Top 5 journals that contributed in producing scientific articles on infertility over the period 2011-2015

**No.**	**Journal**	**Frequency**	**Percentage**
1	Fertility and sterility	1022	15.58
2	Andrologia	291	4.45
3	Journal of assisted reproduction and genetics	212	3.23
4	Human reproduction	203	3.09
5	Reproductive biomedicine online	177	2.69

**Table IV:** Hot topics in the scientific articles in the field of infertility based on betweeness indicators over the period 2011-2015

**Number**	**Infertility area keywords**	**Betweenness centrality**
1	Fertilization in vitro	4,112
2	Adverse effects	4,112
3	Assisted reproductive techniques	4,112
4	Follicle stimulating hormone	4,112
5	Treatment outcome	4,112
6	Therapeutic use	4,112
7	Odds ratio	4,112
8	Biomarkers	4,112
9	Pregnancy outcome	4.112
10	Sperm injections, intracytoplasmic	4.112
11	Age factors	4.112
12	Abortion, spontaneous	3.992
13	Spermatozoa	3,979

## Discussion

As Price mentioned in his 1963 book “Little Science, Big Science”, the number of scientific papers doubles every fifteen years. Such a growth in volume cannot be attributed just to one factor. Therefore, it can be concluded that such growth “is a part of scientific nature” (24). 

Analyses results shows that scientific productions related to infertility which have been indexed on PubMed over the period 2011-2015 have had a growing trend. The growth in scientific documents in other thematic areas during the past years have been confirmed through studies such as the one by Hou *et al* in assessing life cycle (21), cancer (14), diabetes (25), reproductive biomedicine (26) and male infertility (27). It is not surprising that the USA was the leading country in publication output on infertility, a fact that has also been described in other biomedical fields (13, 26-28).

Based on what Garfield has expressed, co-citation scientific maps have been considered as a unique method to study the structure of science thorough which we can establish research structure in specific areas of science (29). Therefore, more scrutiny over infertility maps and creating them in different years can help us identify frequently-used topics. The two areas namely "Adverse Effect" and "Fertilization in Vitro" are ranked first and second respectively among frequently-used thematic fields in PubMed over the research time span. In the maps prepared and considering the indicators of closeness centrality and betweenness centrality, we can conclude that the thematic topics "fertilization in vitro, adverse effects, assisted reproductive techniques, and follicle" are the most valuable from the point of view of both indicators. This finding is consistent with other research (27, 28, 30). Some previous studies have indicated other keywords like "cell, expression”, and “woman" are important (26). Our findings confirm that the MeSH headings generates conceptual keywords. The keyword ‘infertility’ was mostly present in the subject category of assisted reproductive techniques (ART). Time and development of science have both evolved infertility from its primitive status as ART as well as IVF are said to be acceptable methods to treat infertile couples. Nowadays, many infertile couples benefit from IVF since it is considered as the most effective technique which is known as a technique to fertilization in vitro. The IVF is to culture the female egg and male sperm in the laboratory environment and then to transfer embryo into the female's uterine cavity (31). 

After all, none of the treatments can be certified risk-free and the IVF also has some adverse hazards including ovarian hyperstimulation, bleeding, infection, miscarriage, etc. (32). The important point is to recognize the side effects of these treatments. Informing the couples about the possible dangers and risks of using infertility treatment methods is critically important. 

Apart from being linked with many other areas, these thematic fields have a very critical situation in the map in a way that the relationship between many of the topics is formed merely through such thematic fields. In fact, these thematic fields enable the transfer of information in the network. Hence, concepts with high closeness centralities will have specifications such as quick access to other concepts of the network, short distance to other concepts, and high visibility over what is happening in the network. But concepts with high betweenness centralities will generally have features such as the desirable and stable situation in the network, displaying separate breakup points, and considerable effect on what is happening within the network (33).

## Conclusion

This study provided an alternative perspective to the global research trends in infertility studies during the period 2011–2015. Co-word analysis method enables us to represent the structure of internal and external relationships between thematic factors in an objective manner. This can aid us in recognition of the structure of thematic relationships in any area. Therefore, using the results yielded from this research can help us present clear and satisfactory analyses on current situation, research topics and their relationship, and important expressions in the area of infertility. Moreover, users and researches can have a better understanding of thematic and scientific situation in this area and establish directions for further research. We must, of course, take this limitation into account that studied data are from PubMed database. Searching other databases such as Scopus or Web of Science may lead to different results. However, MeSH headings are only available through the MEDLINE and PubMed interfaces. In addition, performing similar researches using other scientometric techniques such as citation analysis, studying co-authorship and creating scientific maps can act as a complement this research.
